# Molecular characterization of fungi causing colonization and infection in organ transplant recipients: A one-year prospective study 

**DOI:** 10.18502/cmm.6.1.2505

**Published:** 2020

**Authors:** Fereshteh Zarei, Seyed Jamal Hashemi, Mohammadreza Salehi, Shahram Mahmoudi, Ensieh Zibafar, Zahra Ahmadinejad, Abbas Rahimi Foroushani, Pegah Ardi, Roshanak Daie Ghazvini

**Affiliations:** 1 Department of Medical Parasitology and Mycology, School of Public Health, Tehran University of Medical Sciences, Tehran, Iran; 2 Food Microbiology Research Center, Tehran University of Medical Sciences, Tehran, Iran; 3 Department of Infectious Diseases, Imam Khomeini Hospital Complex, Tehran University of Medical Sciences, Tehran, Iran; 4 Department of Medical Parasitology and Mycology, School of Medicine, Iran University of Medical Sciences, Tehran, Iran; 5 Liver Transplantation Research Center, Department of Infectious Diseases, Imam Khomeini Hospital Complex, Tehran University of Medical Sciences, Tehran, Iran; 6 Department of Epidemiology and Biostatistics, School of Public Health, Tehran University of Medical Sciences, Tehran, Iran

**Keywords:** Aspergillosis, Candidiasis, Invasive fungal infections, Mucormycosis, Organ transplantation

## Abstract

**Background and Purpose:**

Organ transplant recipients are vulnerable to fungal infections. The aim of this study was to determine the prevalence of fungal colonization and infections among patients who underwent various transplantations and molecularly characterize the etiological agents.

**Materials and Methods:**

This study was conducted on candidates for transplantation in Imam Khomeini Hospital, Tehran, Iran, from April 2017 to April 2018. All patients were monitored for fungal colonization or infections before and after transplantation. Isolated fungi were identified using molecular methods.

**Results:**

A total of 125 patients, including 86 males and 39 females, with the mean age of 52.2 years participated in the study (age range: 15-75 years). Out of 125 patients, 84 (67.2%) cases had fungal colonization that appeared pre- and post-transplantation in 21 and 63 cases, respectively (alone or concurrent with another infection in 55 and 29 cases, respectively). In addition, a total of 39 episodes of fungal infections were diagnosed in 36 (28.8%) recipients (alone or concurrent with colonization in 7 and 29 cases, respectively). Out of the 39 fungal infections, 9 cases appeared pre-transplantation, while the other 30 cases occurred post-transplantation. However, no fungal colonization or infection was observed in 34 (27.2%) patients. Oral candidiasis (n=20) was the most common type of infection, followed by funguria (n=7), onychomycosis (n=5), candidemia (n=3), rhinocerebral mucormycosis (n=1), cutaneous mucormycosis (n=1), cutaneous aspergillosis (n=1), and peritonitis (n=1). Six yeast species were recovered from colonization cases with the dominance of *Candida albicans* both before and after transplantation. The observed fungal infections were caused by 11 distinct species, including the members of *Candida* (i.e., *C. albicans*, *C. glabrata*, *C. parapsilosis*, *C. tropicalis*, and* C. krusei*), *Aspergillus* (i.e., *A. oryzae* and *A. candidus*), *Rhizopus* (i.e., *R. oryzae* and *R. microsporus*), *Trichosporon asahii, *and *Trichophyton interdigitale. *The results also indicated that the development of a fungal infection post-transplantation was associated with fungal colonization (r=0.0184; *P=0.043*).

**Conclusion:**

Based on the results, fungal colonization was a common finding in transplant recipients at Imam Khomeini Hospital. However, the incidence of fungal infections was comparable with those of other centers. As the oral cavity was the most common site of colonization and infection, it might be beneficial to take further care about the oral health of patients using effective mouthwash.

## Introduction

Advances in recent decades in the field of transplantation have resulted in an increase in the number of patients with impaired immune system status [1]. Post-transplant infections still remain a challenge among these patients and can strongly impact the outcome of transplantation [2]. Invasive fungal infections (IFIs) are an important group of post-transplant infections which can lead to high morbidity and mortality rates. As an instance, aspergillosis and candidiasis account for the mortality rates of 65-90% and 30-50%, respectively, among solid organ transplant recipients [2, 3]. The incidence and etiology of IFIs can vary depending on the types of organ transplants and hospital settings [4]. Accordingly, it would be beneficial to perform in-depth studies in all geographical regions and clinical settings in which transplantations is a common practice.

The IFIs are usually caused by *Candida* and *Aspergillus* species. However, other fungi, such as *Cryptococcus* species, members of Mucorales order, and other less common yeast and mold species, can also lead to the incidence of these infections [5]. Reports are suggestive of a changing trend in the etiology of IFIs to previously uncommon species [6], thereby highlighting the need for the utilization of proper identification methods. Furthermore, colonization by fungal species is a risk factor for developing IFIs in vulnerable patients [3]. Accordingly, the surveillance of transplant patients is of clinical importance to prevent morbidity and mortality due to IFIs.

Despite the importance of IFIs in transplant patients, little is known about the epidemiology and etiologic agents of these infections among transplant recipients in Iran [7-12] or the role of colonization in this regard. With this background in mind, the present study was conducted to determine the prevalence of fungal colonization and infections among patients undergoing various transplantations. This study was also targeted toward determining the distribution of various fungal species among these patients using molecular methods.

## Materials and Methods

***Patients and specimens***

This study was conducted on patients who were candidates for transplantation in Imam Khomeini Hospital, Tehran, Iran (as a tertiary care center with an approximate number of 200 transplantations per year) from April 2017 to April 2018. The candidates for bone marrow transplantation were given etoposide, cytarabine, melphalan, and methotrexate. Furthermore, the candidates for kidney transplantation were administered methylprednisolone, a calcineurin inhibitor (mostly tacrolimus), and mycophenolate. Additionally, the lung, liver, or heart transplantation candidates were given prednisolone, tacrolimus, and mycophenolate.

Exposure to antifungal drugs (prophylaxis or treatment) before transplantation was the main exclusion criterion in this study. Clinical specimens, including oral and nasal swabs, as well as urine and sputum samples, were collected from all the participants in two stages, namely pre-transplantation (i.e., one day after admission to hospital), and post-transplantation (i.e., one day after transplantation with two repeats per week until discharge). Nails of all the patients were also screened in both stages, and clippings of clinically suspected nails were collected. For patients suspected of IFIs, blood, bronchoalveolar lavage, tissue biopsy, or peritoneal fluid samples were obtained based on clinical suspicions. All patients were followed up 6 months after transplantation. 

***Laboratory examination of specimens***

Preparations of specimens in 10% potassium hydroxide were examined using a light microscope. Hematoxylin and eosin and gomori methenamine silver stains were also used for the biopsy samples of the patients suspected of having IFIs. Culture was performed on Sabouraud dextrose agar (SDA; Difco, Detroit, USA) plates supplemented with chloramphenicol and incubated at 30°C for a week and checked daily. In case of no growth, the culture media were kept up to 2 weeks before being discarded. Culture of blood samples and other normally sterile body fluids was performed using the BACTEC (Bactec 9120, Becton Dickinson) device according to manufacturers’ instructions. 

Regarding the urine samples, a cutoff of 10^3^ CFU/ml was used to differentiate colonization from infection [13]. For patients with positive urine culture, repeated samples were also collected to confirm the primary result. Observation of pseudohyphae and pyuria in the absence of bacteriuria were confirmatory of infection. 

With regard to the oral samples, the oral cavity of all participants was examined before specimen collection. Positive cultures without clinical symptoms and oral lesions were considered to be colonization. In addition, positive direct examination and culture concurrent with such symptoms as erythema, pseudomembrane in oral mucosa, dry mouth, and glossalgia were regarded as oral candidiasis [14, 15].

***Identification of isolated fungi***

Yeast isolates were subcultured on CHROMagar *Candida* medium (CHROMagar, France) to obtain pure single colonies and perform preliminary species identification. For precise identification, the DNA of yeast isolates was extracted by the simple boiling method [16]. Common *Candida* species were identified by a polymerase chain reaction-restriction fragment length polymorphism  (PCR-RFLP) method on the ITS1-5.8S-ITS2 region of rDNA using *Msp*I enzyme as described previously [17]. For yeasts other than common *Candida* species and *Candida* isolates with inconclusive PCR-RFLP results, ITS rDNA was amplified using the primer set ITS1-ITS4, and the amplicons were sent for sequencing [18]. 

Regarding the mold isolates, DNA was extracted by the phenol-chloroform method [19], and a fragment of ITS rDNA or beta-tubulin gene, depending on the genus of the isolate, was PCR-amplified and sent for sequencing as described previously [20, 21]. Definitive identification of isolates was accomplished by the BLAST analysis (https://blast.ncbi.nlm.nih.gov/Blast.cgi) based on the maximum identities of ≥ 99% and a query coverage of ≥ 98% with verified GenBank sequences.

***Statistical analysis***

Association between baseline characteristics and colonization with fungal infection was analyzed using the Chi-Square test, and *R* coefficient was obtained by logistic regression analysis in SPSS software (IBM SPSS Statistics for Windows, version 21.0; Armonk, NY: IBM Corp).

***Ethical considerations***

This study was approved by the Review Board of Tehran University of Medical Sciences, Tehran, Iran (approval code: IR.TUMS.SPH.REC.1396.3641).

## Results

Totally, 125 patients were included in this study, 44, 40, 30, 10, and 1 of whom were admitted to the bone marrow, kidney, liver, heart, and lung transplantation wards, respectively. The patients were mainly male (n=86, 68.8%), with a mean age of 52.2 years (range: 15-75 years). Out of 125 patients, 84 (67.2%) and 36 (28.8%) cases had respectively fungal colonization (alone and concurrent with another infection in 55 and 29 cases, respectively) and fungal infection (alone and concurrent with colonization in 7 and 29 cases, respectively). Among the infected patients, 3 cases had 2 types of infections (total episodes of infections=39). However, no fungal colonization or infection was observed in 34 (27.2%) cases. Among 29 patients with concurrent colonization and infection, pre-transplantation colonization and post-transplantation infection were caused by the same species in 7 patients.

In the pre-transplantation evaluation, 21 (16.8%) patients had fungal colonization mainly in the oral cavity (n=15), followed by nasal cavity (n=3), simultaneous oral and nasal cavities (n=2), and urinary tract (n=1). *Candida albicans* was the most common colonizer (n=15; i.e., alone and concurrent with *C. glabrata *in 2 and 13 cases, respectively; Table 1). In the post-transplantation stage, 63 patients had new episodes of colonization with the dominance of oral colonization (n=46), followed by urinary tract (n=8), nasal (n=5), and simultaneous oral and nasal (n=4) colonization. *Candida albicans* was the most common colonizer (n=41; alone and concurrent with *C. glabrata *in 34 and 5 cases, respectively). Table 1 shows the prevalence and type of fungal colonization before and after transplantation. 

Before transplantation, 9 patients had fungal infection (5 and 4 cases of oral candidiasis and onychomycosis, respectively). Following transplantation, 26 new episodes of fungal infections were diagnosed in 26 patients. In this regard, one patient developed two different infections post-transplantation, and two patients developed new episodes of infection in addition to their pre-transplantation infections (total number of patients=36, total episodes of infections=39). Oral candidiasis (n=15) was the most common infection. Table 2 presents the prevalence and type of fungal infections in transplant recipients before and after transplantation. 

Following molecular identification, the sequences of the isolates obtained from the cases of colonization or infection that were identified using PCR sequencing were deposited in GenBank (accession numbers: MN944496 to MN944506). Distribution of various fungal species isolated from the episodes of colonization or infection based on the results of molecular methods is shown in Table 3. Regarding the treatment and outcome, three subjects passed away among the patients with fungal infection (Table 4). In statistical analysis of data, infection showed no relationship with gender (r=0.104; *P=0.238*) and age (r=0.176; *P=0.112*). Meanwhile, fungal colonization was associated with the development of a fungal infection (r=0.184; *P=0.043*).

**Table 1.** Prevalence and type of fungal colonization in organ transplant patients before and after transplantation

**Type of colonization**	**Type of transplantation (n)**									
	**Bone marrow** **(44)**		**Kidney** **(40)**		**Liver** **(30)**		**Heart** **(10)**		**Lung** **(1)**	
	**Before**	**After**	**Before**	**After**	**Before**	**After**	**Before**	**After**	**Before**	**After**
Oral cavity	7	16	4	11	2	13	2	6		
Nasal cavity	1	1	1		1	4				
Oral and nasal cavities	1	1			1	2				1
Urinary tract	1	3		4		1				
Total (n)	10	21	5	15	4	20	2	6		1

**Table 2:** Prevalence and type of fungal infection in organ transplant patients before and after transplantation

**Type of infection**	**Type of transplantation (n)**									
	**Bone marrow** **(44)**		**Kidney** **(40)**		**Liver** **(30)**		**Heart** **(10)**		**Lung** **(1)**	
	**Before**	**After**	**Before**	**After**	**Before**	**After**	**Before**	**After**	**Before**	**After**
Oral candidiasis	3	6	1	3	1	5		1		
Funguria		3		3		1				
Candidemia						2				1
Onychomycosis		1	2				2			
Peritonitis						1				
Cutaneous aspergillosis						1				
Cutaneous mucormycosis						1				
Rhinocerebral mucormycosis		1								
Total* (n)	3	11	3	6	1	11	2	1		1

* Three patients had two episodes of infection.

**Table 3:** Distribution and frequency of various fungal species causing colonization and infection in 125 transplant recipients

**Isolation source **	**Species (n)**	
	**Pre-transplantation**	**Post-transplantation**
**Colonization **		
Oral cavity	*Candida albicans* (9) *Candida glabrata* (3) *C. albicans* and *C. glabrata* (2)^a^ *Saccharomyces cerevisiae* (1)	*C. albicans *(26) *C. glabrata* (10) *C. albicans* and *C. glabrata *(5)^a^ *Kluyveromyces marxianus *(*Candida kefyr*) (2) *Candida krusei* (2) *Candida tropicalis* (1)
Nasal cavity	*C. albicans* (3)	*C. albicans *(4) *C. tropicalis* (1)
Oral and nasal cavities (simultaneously)	*C. tropicalis* (1) *C. glabrata* (1)	*C. albicans* (1) *C. glabrata* (1) *C. tropicalis* (2)
Urinary tract	*C. albicans* (1)	*C. albicans* (5) *C. glabrata* (3)
**Infe** **ction ** ^b^		
Candidemia		*C. glabrata* (2) *C. albicans* (1)
Oral candidiasis	*C. albicans* (4) *C. glabrata* (1)	*C. albicans *(9) *C. glabrata* (4) *C. tropicalis* (1) *C. parapsilosis* (1)
Funguria		*C. albicans* (3) *C. glabrata* (3) *Trichosporon asahii* (1)
Onychomycosis	*C. albicans* (1) *C. parapsilosis* (1) *Trichophyton interdigitale* (1) *Aspergillus candidus* (1)	*C. albicans *(1)
Peritonitis		*C. krusei* (1)
Cutaneous aspergillosis		*Aspergillus oryzae* (1)
Cutaneous mucormycosis		*Rhizopus microsporus* (1)
Rhinocerebral mucormycosis		*Rhizopus oryzae* (1)

^a^ Simultaneous colonization by two species

^b^ Three patients had two episodes of infection.

**Table 4:** Characteristics of patients, causative agents, treatments, and outcome of various fungal infections diagnosed pre- or post-transplantation among 125 transplant recipients

**No.**	**Gender/ age**	**Type of transplant**	**Type of infection**	**Before/after transplantation**	**Causative agents**	**Treatment ** ^a,b^	**Outcome**
1	F/63	BM	Oral candidiasis	After	*C. albicans*	FLZ 200 mg/d, 7 days	Cured
2	M/32	Kidney	Oral candidiasis	After	*C. albicans*	FLZ 200 mg/d, 7 days	Cured
3	M/62	BM	Oral candidiasis	After	*C. albicans*	FLZ 200 mg/d, 7 days	Cured
4	F/54	BM	Oral candidiasis	Before	*C. albicans*	FLZ 200 mg/d, 7 days	Cured
5	M/60	Liver	Oral candidiasis	After	*C. albicans*	FLZ 200 mg/d, 7 days	Cured
6	M/67	Kidney	Oral candidiasis	After	*C. albicans*	FLZ 200 mg/d, 7 days	Cured
7	M/48	Liver	Oral candidiasis	After	*C. albicans*	FLZ 200 mg/d, 7 days	Cured
8	M/53	Heart	Oral candidiasis	After	*C. albicans*	FLZ 200 mg/d, 7 days	Cured
9	F/54	BM	Oral candidiasis	After	*C. albicans*	FLZ 200 mg/d, 7 days	Cured
10	M/63	BM	Oral candidiasis	Before	*C. albicans*	FLZ 200 mg/d, 7 days	Cured
11	F/45	Liver	Oral candidiasis	After	*C. glabrata*	FLZ 200 mg/d, 7 days	Cured
12	M/50	Liver	Oral candidiasis	After	*C. glabrata*	FLZ 200 mg/d, 7 days	Cured
13	M/47	BM	Oral candidiasis	After	*C. glabrata*	FLZ 200 mg/d, 7 days	Cured
14	F/32	BM	Oral candidiasis	After	*C. glabrata*	FLZ 200 mg/d, 7 days	Cured
15	M/63	BM	Oral candidiasis	Before	*C. glabrata*	FLZ 200 mg/d, 7 days	Cured
16	M/22	Liver	Oral candidiasis	After	*C. tropicalis*	FLZ 200 mg/d, 7 days	Cured
17	M/45	BM	Oral candidiasis	After	*C. parapsilosis*	FLZ 200 mg/d, 7 days	Cured
18	M/60	BM	Funguria	After	*C. albicans*	FLZ 200 mg/d, 7 days	Cured
19	F/48	Liver	Funguria	After	*C. albicans*	FLZ 200 mg/d, 7 days	Cured
20	F/25	Kidney	Funguria	After	*C. albicans*	FLZ 200 mg/d, 7 days	Cured
21	F/62	BM	Funguria	After	*C. glabrata*	AmB 0.3–1 mg/kg/d, 7 days	Cured
22	F/51	BM	Funguria	After	*C. glabrata*	AmB 0.3–1 mg/kg/d, 7 days	Cured
23	M/43	Kidney	Oral candidiasis	Before	*C. albicans*	FLZ 200 mg/d, 7 days	Cured
			Funguria	After	*C. glabrata*	AmB 0.3–1 mg/kg/d, 7 days	
24	M/67	Kidney	Oral candidiasis	After	*C. albicans*	FLZ 200 mg/d, 7 days	Cured
			Funguria	After	*T. asahii*	VRZ 600 mg twice daily first day, then 200 mg twice daily, 2 days	
25	M/50	Liver	Candidemia	After	*C. albicans*	CAS: 70 mg first day, then 50 mg/d till 2 weeks after first negative blood culture	Cured
26	M/52	Liver	Candidemia	After	*C. glabrata*	CAS 70 mg, 3 days; L-AmB 3 mg/kg/d, 5 days	Died
27	M/38	Lung	Candidemia	After	*C. glabrata*	CAS 70 mg, 3 days; L-AmB 3 mg/kg/d, 5 days	Died
28	M/68	Liver	Peritonitis	After	*C. krusei*	CAS 70 mg first day, then 50 mg/d, 14 days	Cured
29	M/47	Liver	Oral candidiasis	Before	*C. albicans*	FLZ 200 mg/d, 7 days	Cured
			Cutaneous aspergillosis	After	*A. oryzae*	ITZ 200 mg twice daily, 28 days	
30	M/49	Liver	Cutaneous mucormycosis	After	*R. microsporus*	L-AmB 3 mg/kg/d, 28 days	Died
31	F/30	BM	Rhinocerebral mucormycosis	After	*R. oryzae*	L-AmB 3 mg/kg/d, 1 month; POS 400 mg/d, 3 months	Cured

F: female, M: male, FLZ: fluconazole, AmB: amphotericin B, VRZ: voriconazole, CAS: caspofungin, L-AmB: liposomal amphotericin B, ITZ: itraconazole, POS: posaconazole, BM: bone marrow

^a ^Regarding oral candidiasis, caspofungin or amphotericin B was used for cases irresponsive to fluconazole.

^b^ Patients with onychomycosis (n=5; i.e., 4 cases pre-transplantation and 1 case post-transplantation) were recommended to visit a dermatologist after being discharged from hospital; their treatment data are not available and presented.

## Discussion

The present study was conducted to determine fungal colonization and infection in transplant recipients. The results revealed that a large proportion of transplant recipients (63 of 125, 50.4%) were colonized post-transplantation. Furthermore, a notable number of patients (29 of 125, 23.2%) developed new fungal infections, either invasive or non-invasive, after transplantation. 

There are various risk factors for the development of IFI in transplant recipients. In this study, a greater number of fungal infections were observed in patients with colonization (33.3%) than in those without colonization (16.9%). This finding supports the role of colonization as a risk factor for fungal infections, which is in line with other reports [2, 22]. Accordingly, fungal colonization, which has been found to be as high as 45-47.5% among renal transplant recipients [7, 8] and 84% among liver transplant recipients [7] in Iran, should be taken into account. Furthermore, the delayed diagnosis and initiation of therapy for IFIs result in poor treatment outcomes. Patients colonized with pathogenic fungi are the at-risk group in this regard; therefore, fungal colonization should not be overlooked [23]. 

Oral colonization by *Candida* species has been reported in patients with various types of transplantations [7, 8, 24-26], with *C. albicans* being the most common species. In the same vein, our results revealed that oral colonization by *Candida* species was the most common type of colonization, and *C. albicans* was the leading species. The incidence and type of fungal infections may vary depending on different transplantations and geographical regions. Invasive mold infections, particularly aspergillosis, are more common in lung transplant recipients [27]. Marjani et al. [12] reported 8 (40%) cases of aspergillosis among 20 lung transplant recipients. However, the only case of lung transplant recipient in this study passed away because of *C. glabrata* candidemia. 

The dominance of mold infections among hematopoietic stem cell transplant patients has also been reported in different studies [28, 29]. In this regard, Badiee et al. [10] reported 13 (15.8%) cases of aspergillosis among 82 bone marrow transplant recipients. In the current study, there were 10 (25%) cases of non-invasive infections and 1 (2.5%) case of rhinocerebral mucormycosis among 40 bone marrow recipients.

In contrast to lung and bone marrow transplant recipients, among patients receiving other kinds of transplantation (e.g., kidney and liver), candidiasis is the dominant infection [4]. Diba et al. [9] found 41 (32.5%) cases of *Candida* infections among 126 kidney transplant recipients. In this study, 5 (12.5%) cases out of 40 kidney transplant recipients developed a fungal infection, which is lower than the result reported by Diba et al. and similar to the result obtained by Badiee et al. (10.8%) [7]. However, the prevalence obtained in our study is more than the prevalence reported by Honar et al. (2%) [11]. 

Moreover, among 30 liver transplant recipients in the present study, 11 (36.7%) cases of fungal infection were diagnosed, while others reported a prevalence of 12% among these patients [7]. Accordingly, although *Candida* infections were more common than mold infections among liver and kidney transplant recipients, there are notable differences between the prevalence reported in various studies. Therefore, the monitoring of organ transplant recipients for fungal infections should be taken seriously in all transplantation centers. 

In this study, four patients had onychomycosis pre-transplantation, and one case was diagnosed post-transplantation. It has been shown that onychomycosis can be significantly more common in transplantation recipients than in controls [30, 31]. There are also reports regarding the association of onychomycosis with the consumption of tacrolimus among organ transplant recipients [32]. Accordingly, onychomycosis which might not be considered an important infection in transplant recipients should be taken more seriously. This is especially true in patients with onychomycosis due to *Fusarium* species as they might need prophylaxis. In a study on patients with confirmed onychomycosis due to *Fusarium* species [33], it was shown that 4 of 5 patients without anti-mold prophylaxis developed invasive fusariosis while 0 of 6 patients receiving anti-mold prophylaxis experienced this infection. Accordingly, further care should be taken into account for the transplantation candidates who have onychomycosis.

## Conclusion

Fungal colonization was a common finding in the transplant recipients admitted to Imam Khomeini Hospital, Tehran, Iran. In addition, the incidence of fungal infections was comparable with those of other centers. Candida albicans was the leading cause of colonization and infection. As the oral cavity was the most common site of colonization and infection, it might be beneficial to take further care about the oral health of patients using effective mouthwash.
